# Effects of germination and lactic acid fermentation on nutritional and rheological properties of sorghum: A graphical review

**DOI:** 10.1016/j.crfs.2022.04.014

**Published:** 2022-05-10

**Authors:** Melissa Rodríguez-España, Claudia Yuritzi Figueroa-Hernández, Juan de Dios Figueroa-Cárdenas, Patricia Rayas-Duarte, Zorba Josué Hernández-Estrada

**Affiliations:** a Tecnológico Nacional de México/Instituto Tecnológico de Veracruz, M.A. de Quevedo 2779, Col. Formando Hogar, CP 91897, Veracruz, Veracruz, Mexico; b CONACYT-Tecnológico Nacional de México/Instituto Tecnológico de Veracruz, Unidad de Investigación y Desarrollo en Alimentos, M. A. de Quevedo 2779, Veracruz, Ver, C.P. 91897, Mexico; c Centro de Investigación y de Estudios Avanzados del IPN (CINVESTAV Unidad Querétaro), Libramiento Norponiente 2000, Fracc. Real de Juriquilla, Querétaro, C.P. 76230, Qro., Mexico; d Robert M. Kerr Food and Agricultural Products Center, Department of Biochemistry and Molecular Biology, Oklahoma State University, Stillwater, OK, 74078, USA

**Keywords:** *Sorghum bicolor*, Germination, Fermentation, Rheological properties, Gluten-free bread

## Abstract

Sorghum (*Sorghum bicolor*) is a nutritional grain considered an important source of micro- and macro-nutrients. Also, the flour obtained from sorghum is considered a suitable substitute for wheat flour for celiac disease patients due it is gluten-free. However, its use has some limitations due to anti-nutritional factors such as tannins, phytates, trypsin inhibitors, and protein crosslinkers. To prevent those effects, new strategies for sorghum processing have been explored. Germination of this grain has been shown to increase nutrient content further and reduce anti-nutrients. In addition, fermentation with lactic acid bacteria could modify starch and protein digestion in sorghum flour and increase their nutrient availability. Although there are many benefits to germination and fermentation, more research must be done to improve the products' texture and sensory properties to gain wider consumer acceptance. In this review, the mechanism behind changes in the nutritional and anti-nutritional profile of sorghum grain due to germination and fermentation treatments is shown, and the impact of these changes on dough rheological properties and bread quality.

## Highlights

• Sorghum is an important crop due to its agricultural and nutritional characteristics.
• Sorghum has anti-nutritional factors that negatively affect its nutritional value.
• Germination reduces the anti-nutritional factors of sorghum.
• Sorghum fermentation enhances its nutritional, rheological, and sensory properties.

## Graphical abstract

**Figure:**
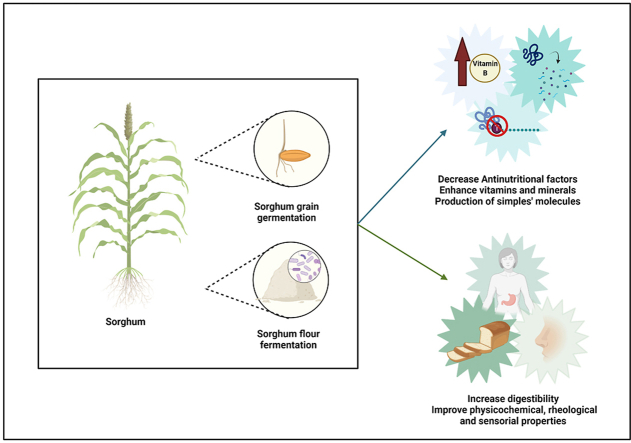

## 1 Introduction

Sorghum is one of the most important cereals in the world after wheat, corn, rice, and barley. It has low water requirements (400–600 mm per year), resists hydraulic stress, drought resistance, and is classified as a C_4_ plant. Like a C_4_ plant, sorghum has higher rate of photosynthesis and agricultural production potential than other crops. Also, it is more resistant to inhibition by oxygen and has lower photorespiration (Serna-Saldivar, 2010; Abah et al., 2020).

In Africa and Asia, sorghum is used as a key base for traditional foods and beverages (e.g., traditional beers, wine, and non-alcoholic beverages) using processes that have been industrialized at a large scale. Sorghum nutritional composition has been reported as an important source of energy, proteins, carbohydrates, vitamins, minerals, and phenolic and flavonoids compounds, which are beneficial for human consumption and are attributed with potential health-promoting effects, such as anti-carcinogenic, antimicrobial, and antioxidant properties. The implementation of different technologies like milling, fermentation, malting, distilling-related technology, among others, has been explored and applied in the development of different food systems such as cookies, bread, noodles, and tortillas. In addition, the lack of gluten proteins makes it a desirable substitute for patients with celiac disease or allergies related to wheat-based products (Smolensky et al., 2018; Aruna and Visarada, 2019).

**Fig. 1:**
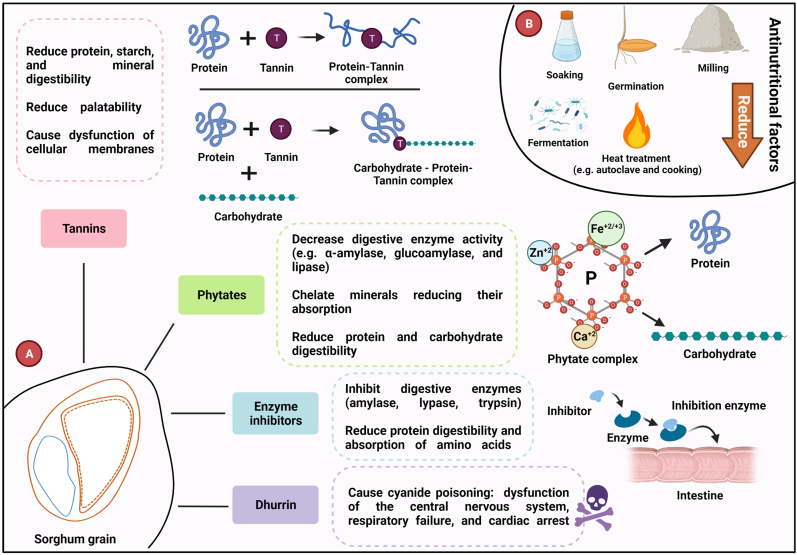
Sorghum anti-nutritional factors. (A) Sorghum anti-nutritional factors. (A) Mainly anti-nutritional factors in sorghum grains that impact human nutrition, negatively affect other metabolic pathways, form interaction complex that reduces the digestibility of carbohydrates and proteins, and hinder bioavailability and absorption of minerals like minerals Fe^+2^/^+3^, Ca^+2^, Mn^+2^, Mg^+2^, and Zn^+2^. (B) Processes such as milling, germination, fermentation, soaking, and heat treatment can help to reduce the concentration of these anti-nutritional factors and enhance the bioavailability of other nutrients.

However, like legumes and seed oils, some compounds affect the nutritional value of sorghum, such as the presence of anti-nutritional factors (Fig. 1). For example, tannins are water-soluble phenolic metabolites that can be classified in three groups: hydrolyzable (e.g., gallitannins and ellagitannins), condensed (e.g., proanthocyadinnins), and complex. Condensed tannins can bind and precipitate protein fractions, rendering them indigestible and limiting their availability. Also, tannis can inhibit hydrolytic enzymes such as trypsin, α-amylase, glucoamylase, and lipase, and bind not only proteins but also minerals and vitamins, rendering them unavailable (Hodges et al., 2021). Phytic acid or phytate is the principal storage form of phosphate, which chelates mineral cations and proteins, forming insoluble precipitated complexes, which leads to reduced bioavailability of trace minerals and reduced digestibility of proteins (Afify et al., 2011). Enzyme inhibitors, such as protease, trypsin, and chymotrypsin inhibitors, are anti-metabolic proteins related to the inhibition of digestible enzymes, which interfere in protein digestibility and hinder the absorption of amino acids (Chhikara et al., 2018). Dhurrin is a cyanogenic glucoside synthesized by three genes (CYP79A1, CYPE1, UGT85B1), and located mainly in the aerial shoot of sorghum plant when ingested and hydrolyzed frees hydrogen cyanide (HCN) causing cyanide toxicity. HCN cause dysfunction of the central nervous system, respiratory failure, and cardiac arrest. Also, excess cyanide ions can quickly produce anoxia of the central nervous system through inactivating the cytochrome oxidase system resulting in death (Gleadow et al., 2021).

## 2 Sorghum germination

**Fig. 2:**
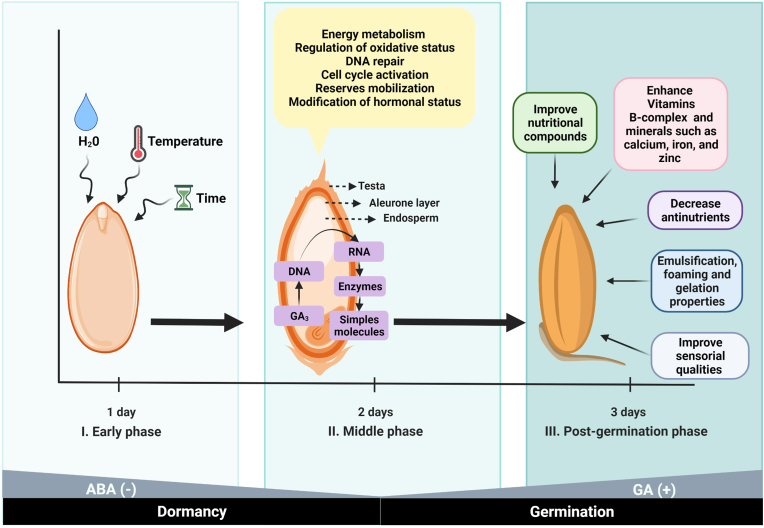
Three stages of sorghum germination process. I. The early phase is defined as the seed imbibition and the early plateau phase of water uptake. The seed development is regulated by water addition and temperature control, but mainly by the effects of chemical factors such as abscisic acid (ABA) and gibberellin (GA). For the release of seed dormancy and subsequent germination, ABA (enhances dormancy and inhibits germination) decreases, while GA (reduces dormancy and promotes germination) increases. II. The middle phase is completed by visible radicle protrusion through the seed covering layers, followed by the seedling establishment. During this phase, many physical and chemical reactions include the rupture of testa, leakage of cellular solutes, repair of organelles, membranes, and DNA, metabolism activation and synthesis of DNA, RNA, and compounds such as proteins and carbohydrates. And finally, in the later phases, seedling development changes the food's nutritional, biochemical, and sensory characteristics enhance desirable flavor and taste.

Germination is the process that occurs at the beginning of the development of seeds into plants (Fig. 2). It is a process widely used in legumes and cereals changing their nutritional, biochemical, and sensory characteristics, enhancing desirable flavor and taste. These changes are associated with the activation of some enzymes like amylases, proteases, phytases, lipases, and fiber-degrading, leading to the breakdown of proteins, carbohydrates, and lipids into simple molecules; contribute to the partial hydrolysis into glucose, maltose, and a wide variety of dextrin's starch, thereby increasing the content of sugar. Germination reduces anti-nutritional factors resulting in higher in nutritional quality than non-germinated seeds (Zhang et al., 2015).

In several sorghum samples, germination decreases the content of proteins (12–21% on average) but increases protein solubility and digestibility. This is due to a high proteolytic activity of storage proteins and increase of their bioavailability and content of amino acids such as lysine, valine, phenylalanine, methionine, and tryptophan. In addition, a significant increase in the nitrogen solubility index is also found, which might be due to the degradation of proteins into amino acids and short peptides (Afify et al., 2012a).

Carbohydrates changes showed significant increase in total soluble sugar, reducing sugars, and damaged starch, while decreasing starch due to the increased activity of α-amylase that hydrolyze starch into smaller sugar molecules (Dicko et al., 2006). Lipid content or crude ether extract decrease (13–21% on average) after germination except for some sorghum cultivars. The reduction of fat content may be due to biochemical and physiological changes during germination that utilized fat as energy (Afify et al., 2012b). During germination some B-vitamins increase, such as riboflavin, while the concentration of total thiamine and total pyridoxine decrease. However, some authors have reported that germination did not affect the concentration of vitamins, including vitamins of B-complex such as folic acid, niacin, thiamin, and pyridoxine, while vitamin E was decreased (Pinheiro et al., 2021). Macro-elements (phosphorus, potassium, magnesium, sodium, and calcium) content decreased after germination, including micro-elements (iron, zinc, manganese, and copper) content. However, the bioavailability of calcium, iron, and zinc increased significantly in most varieties and cultivars of sorghum because the anti-nutritional factors like phytic acid are reduced (Afify et al., 2012c).

In sorghum, germination has decreased tannin, phytic acid, and oxalate content. The decrease in tannin content was explained as hydrogen bonding and nonpolar hydrophobic interactions of tannin and proteins. The phytic acid decrease was attributed to leaching in the sprouting medium and increased activity of polyphenol oxidase and other catabolic enzymes. Germination of sorghum grains increased hydrogen cyanide attributed to the synthesis of dhurrin from amino acid precursors such as tyrosine during sprouting (Mohamed Nour et al., 2010; Ojha et al., 2018).

## 3 Sorghum lactic acid fermentation

**Fig. 3:**
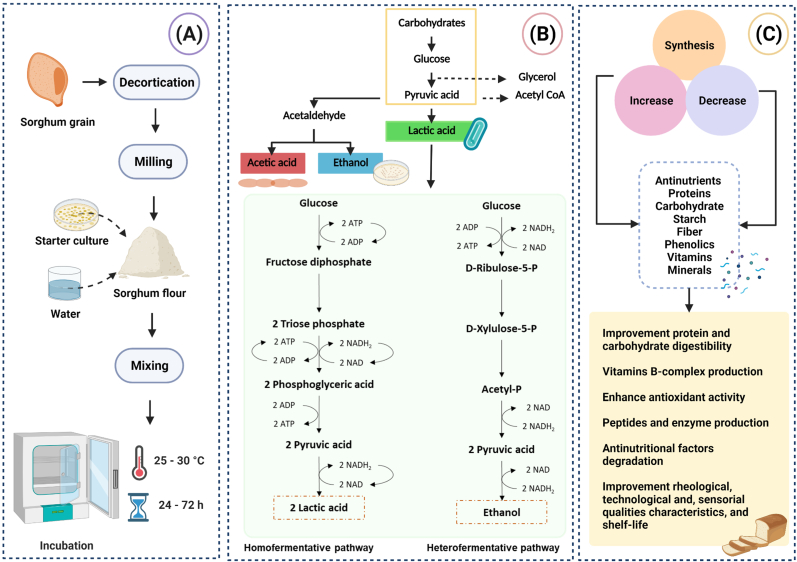
(A) Sorghum fermentation process. The grain is decorticated and milled to obtain the flour, fermented. Subsequently, (B) sorghum fermentation can be classified into an alcoholic, lactic acid, and acetic fermentation. Alcoholic fermentation produces ethanol by yeast. LAB strains mainly perform lactic acid fermentation in two main ways: Homofermentative (a process that ferments lactose into pyruvic acid, which is then reduced to lactic acid) and heterofermentative (a process characterized by the formation of co-products such as CO_2_, ethanol, and acetic acid in addition to lactic acid); and finally acetic fermentation carry out by acetic acid bacteria to convert alcohol to acetic acid in the presence of oxygen. According to inoculation and fermentation conditions, these processes can be cataloged as spontaneous, back-slopping, and controlled fermentation. (C) Microbial activity during sorghum fermentation can lead to the synthesis of several compounds such as organic acids that can inhibit and suppress the growth of pathogenic and spoilage microorganisms. Also, anti-nutritional factors can be reduced during fermentation, and nutritional, rheological, technological, and sensory characteristics such as flavor, texture, and aroma in products made from this cereal can be improved. During this process, the activation of various enzymes (e.g., amylases, xylanases, hemicelluloses, and proteases) triggers the breakdown of large biomolecules, allowing the synthesis/release of various bioactive compounds, which have antimicrobial, antioxidant, immunomodulatory, and other beneficial effects.

Fermentation is one of the oldest food processes and can be defined as the intentional substrate changes into new products/forms through microbial action. This process has been related to improving nutritional, rheological, and sensory qualities due to enzymatic activity caused by microbial actions in the reduction of the size of molecules and production of new compounds. Lactic acid fermentation is one of the most common forms of fermentation in sorghum (Fig. 3). This fermentation carried out by lactic acid bacteria (LAB) inhibits other microorganisms through the utilization of available carbohydrates and accumulation of organic acids mainly lactic acid and other metabolites in the food matrix mainly through glycolysis, lipolysis and proteolysis. LAB strains contribute to the enrichment of nutrients such as B-complex vitamins, as well as improvement of flavor, and texture, due to the synthesis of exopolysaccharides (EPS), lipids, and proteins, and reduction in anti-nutritional factors (Adebo, 2020).

LAB enzymatic hydrolysis significantly increases the bioavailability of protein and available amino acids such as lysine, leucine, isoleucine, and methionine, which can benefit the host's nutritional status, particularly if the host has a deficiency in endogenous protease production. Also, increase of protein solubility is influenced by processing conditions, pH, and ionic strength. This can be attributed to structural changes in protein by the fermentation and inactivation of anti-nutritional factors like phytates (Abd Elmoneim et al., 2005).

Carbohydrates decrease with fermentation since there are the main source of nutrients required for the glycolysis pathway but increase starch bioavailability and digestibility due to the reduction of anti-nutritional factors. In the same way, a significant decrease in fat content has been associated with the biochemical and physiological changes, like the increased activity of lipolytic enzymes, which hydrolyze triglycerides to fatty acids and glycerol (Simwaka et al., 2017).

During fermentation, phenolic compounds classified into two categories: phenolic acids (benzoic or cinnamic acid) and flavonoids (tannins and anthocyanins), are metabolized and modified into other conjugates, glucosides, and related forms resulting in the activation of metabolism of LAB strains. In sorghum, phenolic compounds such as catechin, gallic acid, and quercetin increase after fermentation. However, the content of flavonoids, total tannins, and total phenolic compounds decrease. It has been reported that LAB fermentation led to the modification of phenolic compounds into structurally related compounds. All of these changes were attributed to decarboxylation, hydrolysis, and esterification during fermentation (Adebo et al., 2018).

The effect of sorghum fermentation using LAB strains on anti-nutritional factors confirmed a significant decrease in anti-nutritional factors such as tannin and phytates due to tannase and tannin acyl hydrolases that break down tannin complexes and phytase activity that degrades phytate. Other compounds like oxalate and dhurrin are also significantly reduced and their decreases have been associated with the hydrolysis of certain compounds, low pH, and microbial activity (Simwaka et al., 2017; Ojha et al., 2018).

## 4 Rheological, technological, and sensorial changes in sorghum dough germinated and fermented

**Fig. 4:**
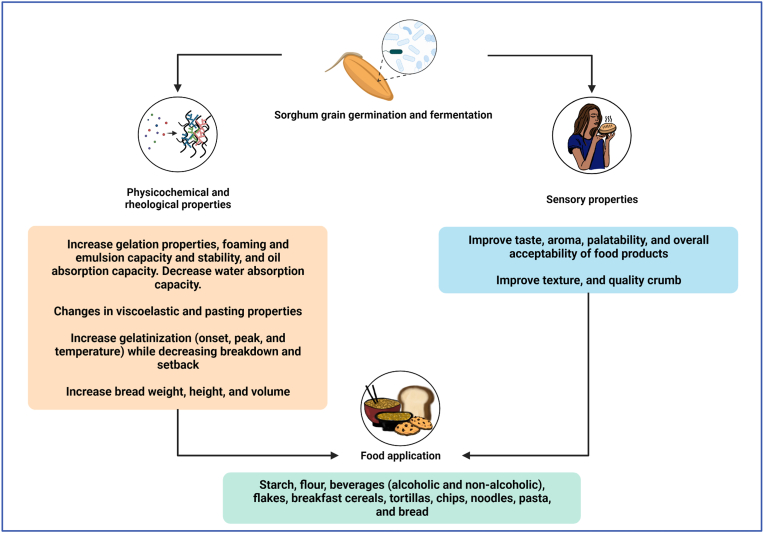
Changes in the rheological and sensorial properties in the germinated and fermented sorghum grain and potential functional foods can be used. Decomposition of the high molecular weight polymers during the germination and fermentation process leads to the generation of bio-functional substances and the improvement of the rheological and sensorial qualities due to softening of texture and enhancing flavor. These changes have been attributed to metabolic actions and products of the fermenting microbiota and endogenous flour enzymes (amylase and protease). Sorghum is consumed in a wide variety of traditional foods. Sorghum-based food products have the potential to be incorporated in acceptable foods with health benefits, consumer acceptability, and commercial value specially in gluten-free products for the general public and patients with celiac disease or gluten sensitivity.

In addition to the nutritional changes in the sorghum grain carried out by germination and fermentation, changes in the rheological, technological, and sensorial properties have been reported (Fig. 4). As proteins increase their solubility during the germination and fermentation process due to the low pH, it promotes intramolecular electrostatic repulsion, making protein groups more reactive (denaturation) and causing more aggregation. The aggregation of proteins in the liquid phase of the sorghum batter during baking can result in weaker structure and irregular crumb structure (with a flat top and holes) attributed to repulsion at the molecular level and interference with starch gelatinization. The result contributed to high resistance to deformation of starch gel resulting in a more desired texture of sorghum products (Olojede et al., 2020).

It has also been reported that low pH causes depolymerization (break of glycosidic bonds of the starch molecules) and partly inactivates amylases, especially α and β-amylases, and increases the solubility of cellulose and hemicellulose (Hugo et al., 2003).

The enzymatic actions generated in dough fermentation using LAB strains result in slight changes, mainly in granule morphology, high loaf volume, more evenly distributed gas cells with thinner walls between, and starch granule swelling. Some LAB strains, such as *Leuconostoc mesenteroides*, have a positive effect on the viscoelastic properties of dough due to the production of EPS like dextran, which improve baking properties such as texture and volume. Prominent among these benefits is improving dough stability, texture, and bread acceptability. Fermentation enables EPS synthesis increasing the sorghum batter's elastic component (Edema, 2011).

The interactions between proteins, starch, and lipids are important in the formation of viscoelastic properties in the dough. In the physicochemical properties of sorghum, an increase in gelation properties has been observed related to a good gel-forming or firming agent and emulsifying capacity and stability due to the positive correlation between hydrophobicity and protein stability. The foaming capacity and foaming stability also increased due to the concomitant increase of solubilization and denaturation of proteins while reducing the molecules' surface tension, which improves dough formability. Reports of increased oil absorption capacity and water absorption capacity during germination were attributed to protein and polysaccharides breakdown, this contributes to a decrease of the amount of water available for gelatinization (Abd Elmoneim et al., 2005; Elkhalifa and Bernhardt, 2010).

In rheological properties, it was observed a decrease in the elastic moduli $\left(G^{\prime }\right)$, loss moduli $\left(G^{\prime \prime }\right)$ and complex viscosity (η∗), which means that the viscous properties of the batters increased while the elastic behavior decrease. However, if during the EPS production by LAB strains, the $G^{\prime }$*,* $G^{\prime \prime }$ and η∗ increase, they contribute to an increase resistance to deformation of the batter. In addition, a significant decrease in breakdown and peak viscosity was attributed to the degradation of protein and starch. Due to the aggregation of amylose molecules, a decrease in final viscosity and setback was also observed. An increase in viscosity while a decrease in swelling factor and pasting temperature was also observed, and were influenced by the enzyme activity, specific amylase, and protease, on protein, lipid, and starch (Li et al., 2020; Olojede et al., 2020).

## 5 Conclusions

Sorghum grain is a promising gluten-free alternative to wheat flour for the development of foods. This review discusses some effects on the nutritional, anti-nutritional, physicochemical, rheological, and sensory properties of sorghum grain after germination and fermentation processes. More research is needed in areas of germination and fermentation by potential strains of LAB for improvement of the nutritional quality of the products. Additional studies are also needed in characterizing the physicochemical and rheological properties of the optimized germinated/fermented grains and their products, as well as their implementation in gluten-free products.

## CRediT authorship contribution statement

**Melissa Rodríguez-España:** Conceptualization, Investigation, Writing – original draft, Visualization. **Claudia Yuritzi Figueroa-Hernández:** Conceptualization, Supervision, Writing – review & editing. **Juan de Dios Figueroa-Cárdenas:** Writing – review & editing. **Patricia Rayas-Duarte:** Writing – review & editing. **Zorba Josué Hernández-Estrada:** Conceptualization, Supervision, Writing – review & editing.

## Declaration of competing interest

The authors declare that they have no known competing financial interests or personal relationships that could have appeared to influence the work reported in this paper.
